# “It is painful and unpleasant”: experiences of sexual violence among married adolescent girls in Shinyanga, Tanzania

**DOI:** 10.1186/s12978-020-01058-8

**Published:** 2021-01-02

**Authors:** Christine Mwanukuzi, Tumaini Nyamhanga

**Affiliations:** 1 United Nations Population Fund, Dar es Salaam, Tanzania; 2 Department of Development Studies, Muhimbili University of Health and Allied Sciences, Dar es Salaam, Tanzania

**Keywords:** Sexual violence, Painful sex, Non-consensual sex, Rape, Married adolescent girls

## Abstract

**Background:**

While the situation of married adolescent girls in Tanzania is increasingly documented, empirical evidence concerning the ways in which child marriage impacts girls’ and young women’s sexual lives is limited. Specifically, little is known about lived experiences on sexual violence among married adolescent girls in Tanzania.

**Methods:**

This article reports on a qualitative study using a phenomenological approach to describe married girls’ experiences of sexual violence in the Shinyanga Region, an area with the highest prevalence (59%) of child marriage in Tanzania. Data were collected from 20 married girls aged 12–17 years.

**Results:**

The study identified four analytical themes regarding the experience of sexual violence, namely: forced sex; rape; struggling against unpleasant and painful sex; and inculcation of the culture of tolerance of sexual violence.

**Conclusion:**

The study highlights the voices of married adolescents on an important but a neglected topic of relevance to Tanzania’s public health. Findings from this study suggest that married adolescent girls suffer sexual coercion in silence.

**Plain English summary:**

Child marriage is a major public health problem in sub-Saharan Africa in general and in Tanzania in particular. However, there is limited research on the ways in which it impacts sexual lives of married adolescent girls. In response to the inadequacy of information, married adolescent girls in Shinyanga Region of Tanzania were requested to voice out their experiences of sexual violence. Three themes were identified from the responses, namely: forced sex; rape, struggling against unpleasant and painful sex; and the inculcation of the culture of tolerance of sexual violence.

In conclusion, this study has echoed voices of married adolescent girls on the sexual troubles they experience. Their main concern is that they suffer sexual coercion in silence, which increases their risk of acquiring sexually transmitted infections and/or unwanted pregnancies. Recommendations for sexual violence prevention strategies are discussed.

## Introduction

Child marriage is a serious problem in Africa, Asia and Latin America [1–4]. The prevalence of child marriage in Tanzania is estimated at 37%; with majority of the victims in rural areas [5]. Factors contributing to child marriage include gender inequality, marriage-related social norms pregnancy during adolescence, poverty, and poor enforcement of laws [5]. In addition, the consequences of child marriage include but are not limited to gender-based violence, sexually transmitted infections, dropping out of school, and adolescent pregnancies [5, 6]. Compared with women aged 20–24, pregnant adolescents have a higher risk of developing obstetric complications such as eclampsia, systemic infections and pre-term birth [7]. Indeed, Adolescents younger than 15 years are more likely to die from these obstetric complications [8–10].

Furthermore, marriage to a much older man coupled with masculine societal norms of domination, makes adolescent girls more likely to be the victims of intimate partner violence [11]

Intimate partner violence (IPV) is common in sub-Saharan African countries [12–15]. An analysis of Demographic and Health Survey (DHS) data in eight African countries (Malawi, Zambia, Zimbabwe, Kenya, Rwanda, Burkina Faso, Mali, and Liberia) indicated that 20–50% of married women report having experienced IPV [16, 17]. Unacceptably high levels of physical and sexual violence against women have also been reported in Tanzania. For example, in 2005 the World Health Organization published a report showing that 41% of ever-partnered women in Dar es Salaam and 56% in Mbeya had ever experienced physical or sexual violence perpetrated by a male partner [18]. McCloskey [19] reported that 21% of women in Moshi experienced physical and/or sexual violence. Stocklet al.[20] report that 33% of young women aged 15 to 24 in Tanzania have ever experienced IPV.

Although the above evidence is suggestive of IPV in Tanzania [18, 19, 21–23], specific evidence on married adolescent girls is not yet definitive despite higher vulnerability to to non-consensual sex and attendant sexual and reproductive health consequences [24–30]. Likewise, IPV continues to receive relatively little attention from Tanzania’s researchers, policy makers and programme managers in Tanzania [31, 32]. This current study describes the sexual concerns of married adolescent girls in Shinyanga – a region with the highest level of child marriage in Tanzania [33], thereby contributing to the evidence of IPV among married adolescents in the region.

## Methods

### Design

A qualitative phenomenological design, was employed to explore married adolescent girls’ experience of sexual violence.

### Study setting

The study was conducted in Kahama District in the Shinyanga Region of Tanzania, during July 2015. While child marriage is common in almost all regions of Tanzania, the prevalence of this practice in Shinyanga is 59%, which is the highest in the country [33]. Kahama District was selected since it has the highest level of child marriage within the region—as reported by the programme manager of a Non-Governmental Organization (NGO) supporting married adolescent girls in the area (Personal communication, May 12, 2015).

### Sampling

With the assistance of the NGO staff, a purposive sampling strategy was used to recruit 20 married adolescent girls between the age of 12–17 The sample size was determined during fieldwork on the basis of saturation [34–36].

### Data collection

Data were collected through in-depth interviews conducted over a two-week period. The method enabled married girls to tell stories of their lived experiences. The interviews were conducted by the first author (CM) and rapport was developed in four ways: firstly, the respondents had prior familiarity with the interviewer; secondly, the venue for the interviews ensured privacy; thirdly, consent was sought after an explanation was provided on what the study aimed to achieve and each respondent had been assured of confidentiality; and fourthly, the interviewer conducted interviews in a manner that showed interest and concern for the respondent’s circumstances. An interview guide that consisted of open-ended questions was used to collect information on lived sexual experiences. However, questioning did not follow a fixed order [35] but varied in response to interaction with the informants. Interviews were conducted in Kiswahili, tape recorded and transcribed verbatim. Each interview lasted an average of 45 min.

### Data processing and analysis

Data were analyzed manually using thematic analysis [37, 38]. Analysis began by an initial line-by-line coding of field notes and transcripts, resulting in 58 codes. This was followed by an in-depth examination of the resultant codes and their categorization into eight descriptive themes. These were further condensed into four abstract analytical themes around which the results are presented.

## Results

### Participants’ socio-demographic characteristics

The ages of the study participants were between 12 and 17 years (Table 1). About one third of them were between 12 and 14 years of age. Thirteen adolescent girls reported to have an age gap of between 30 and 40 with their spouses. Of the 20 participants, 11 girls had completed primary education, four had dropped out at lower primary school, and five had gone to secondary school but later dropped out of school. All participants reported to have been forced into marriage, some by their parents, so they could receive a dowry, and some by poverty within their parental families.

**Table 1:** Sociodemographic characteristics of respondents

Characteristics	Number (n = 20)
Age (years)	
12–14	6
15–17	14
Age difference with spouses	
30–40 years	13
More than 40 years	7
Marital status	
Widowed	0
Divorced	3
Married	17
Education level	
No formal education	0
Primary incomplete	4
Primary complete	11
Secondary incomplete	5

The participants’ lived experiences are organized within the following four themes: non-consensual sex; rape; struggling against unpleasant and painful sex; and inculcation of the culture of tolerance of sexual violence.

### Non-consensual sex

Participants asserted that by being young they had limited power to negotiate with their much older spouses whether, when and how to have sex. Their partners pressurized them—through verbal threats and even beating–to having sex even when they did not want to. One 15-year-old girl married to a 46-year-old man said that she could not even think of saying no to sex when her husband demanded it. Although the husband had three other wives and visited her home once in a while, he expected her to be ready for him at any point in time:

*He always come home for sex. He expects me to be ready. Even when I am tired I can’t refuse or even indicate the fatigue. He will beat me. He also gives me little amount of money when he wants. I can’t ask. If I have a need for money I go to my mother. ((Married adolescent girl, 15 years)*

When married, girls are expected to immediately adapt to their new roles as wives and be able to fulfil their husbands’ sexual needs and desires. Husbands of some of those who demonstrate not being ready for sex are often not patient enough to wait but coerce their young wives to have sex through threats and intimidation. Another respondent said:

*He often forces me to have sex. Even when I say I am not ready, he does not respect my position, he threatens to beat me up and lock me out of the room. (Married adolescent girl, 12 years)*

Some respondents reported their sexual initiation in marriage was characterized by use of physical force which traumatized them and caused significant bleeding, a 15-year-old girl married to a 46-year-old man, recalled her experience thus:

*I bled a lot and was sick in bed for a whole week. No one took me to hospital. Instead, I was warmed up with hot water and given a lot of fluids to drink. I can’t forget the experience. It was so bad and still am not enjoying [sex]. (Married adolescent girl, 15 years)*

It was reported that when married adolescent girls express fear and reluctance to participate in sex, they are taken as being disobedient. Hence, men force themselves upon them and the girls are expected to accept it and get used to it over time.

## Rape

All sexual encounters with respondents aged between 12 and 14 years of age, not only that they were non-consensual but also amounted to rape. This is because those adolescents were below the minimum legal age of marriage, which is 15 years of age—as per the Sexual Offences Special Provisions Act (SOSPA) number 4 of 1998 [39]. Section 130 (2) A of the SOSPA, for example, states:

*A male person commits the offence of rape if he has sexual intercourse with a girl or a woman in the circumstances falling under the following descriptions: e) with or without her consent when she is under 18 years of age, unless the woman is his wife 15 or more years of age and is not separated from him.*

### Struggling against unpleasant and painful sex

Some of the respondents feared having sex either because their first experience was painful, or they did not expect sex to be a routine exercise and even worse, they could not communicate their worries to their spouses. Most of them had to persevere with the pain although few were lucky to be listened to after expressing themselves by crying or by being numb. A girl aged 16 years said it took her two months of ‘counselling’ by her sisters n-law to accept sleeping with her husband. She said:

*It was my first time in life to sleep with a man on the same bed the whole night! I could not imagine that, and he seemed not to care and made advances on me. So, when he entered the room, I would scream loudly, and he would leave quickly. That was my protection. I think then my mother-in-law heard this and talked to her daughters. (Married adolescent girl, 16 years)*

Another girl refused to continue having sex with her husband because she felt that it was not pleasant and was painful. She conceived the first time she had sex and thereafter, used her pregnancy as her defense not to have any more sex. However, her husband tried to persuade her, but she continued to refuse by crying out loudly until her husband decided to leave her and he never went back to her.

*I do not care if he comes or not and I do not want any other man, ever. I do not need sex. I have my child and that’s enough for me.(Married adolescent girl, 14 years)*

Ever since, she reported, life became difficult so much so that she had to move and live with her elder sister. Her son is now one year old, and the father has not been in contact with the family. The study also found that, since many of these married adolescent girls were not yet psychologically ready for sex, they have never enjoyed it. They narrated that often times they engage in sex as a duty to their husbands because they cannot refuse and cannot express their discontent.

### Inculcation of the culture of tolerance of sexual violence

The married adolescent girls also expressed a concern that culture and the teachings from their parents and guardians render them incapable of negotiating sex. When asked if they can say no to sex, some said they can, though to most girls, such a question was shocking. One participant asked the researcher, “How can you refuse your husband such a thing? It is his right. What would I have done to make me tired and refuse?!” She could not imagine such an option even existed. The training many had from their aunts, mothers or grandmothers, as a preparation for their weddings, was an emphasis on being submissive to their husbands and especially not refusing to have sex upon their husbands wishes.. Another girl narrated that her husband had abandoned her during her pregnancy due to fear of the responsibility and she was left with her husband’s grandmother. When the husband came back after she had delivered, she had to sleep with him without consenting:

*He is my husband and I shouldn’t question him especially when it comes to sex. It is against the teaching I got. In our culture, we believe that sex holds the marriage and no woman should deprive her husband that right. (Married adolescent girl, 17 years)*

### Trustworthiness

The degree to which the study findings can be trusted [trustworthiness] was achieved by implementing strategies for attaining three quality criteria in qualitative research, namely: credibility, dependability and transferability [40]. Several ways were used to achieve credibility of the results. Firstly, the interview guide was piloted to improve the questions and the interviewing skills. Secondly, a member check technique was applied during interviews whereby the interviewer summarized the information from the interviewee. Thirdly, this paper benefited from investigator triangulation whereby, both authors took part in data analysis, both independently and jointly. The findings presented in this paper arose from the consensus between the two authors. Therefore, both authors are confident that the study findings are valid.

Regarding dependability, the authors are confident that the qualitative findings of this study are logical and grounded in the data. Systematic procedures of conducting in-depth interviews were followed. Questions in the interview guide were derived from the study objectives. Moreover, transferability—which is about applicability of the findings to other settings—was ensured by providing a ‘thick description’ of the respondents and the research process, so that the reader can make judgement whether the findings are transferable to their own setting.

## Discussion

Thus study yielded lived experience on sexual violence among married adolescent girls’ in Tanzania. The interviews identified varied experiences thematically structured across the areas of forced sex; rape, a struggle against painful sex; the culture of tolerance of sexual violence; and lack of protection by the existing laws. The discussion is structured around these areas.

### Non-consensual sex and related implications

This study found that male partners pressurize their young spouses into having sex even when they do not want to. Verbal threats and/or physical beatings are used to force the married adolescent girls into submission. These findings demonstrate married adolescent girls’ heightened vulnerability to all forms of violence; sexual, emotional and physical. The male partner’s demand for sexual intercourse seems to be the starting point of disagreement which culminates in violence. That is, if an adolescent girl refuses her husband’s demand for sexual intercourse, the husband would start scolding her, threatening to beat her up and if the girl continues to resist, she may actually be slapped. Although, generally masculine norms encourage married men to force their spouses into non-consensual sex, the married adolescent girls are more susceptible because of the age gap. As Cáceres [41] found in Peru, norms for masculinity in Tanzania include those socio-cultural expectations which promote men’s superiority and dominance over women and women’s submissiveness, as well as those which present men’s sexual desires as uncontrollable and that the wife has to respond to such desires [38]. These findings are corroborated by those reported in other developing countries [24, 27, 42]. Moreover, the findings of the current study illustrate power dynamics in sexual relationships—whereby the much older and physically powerful male partners pressurize their young spouses into sexual submission –as propounded by the theory of gender and power [43].

### Unpleasant and painful sex

The married adolescent girls who participated in this study reported that they do not enjoy sex instead, they only feel pain because of the emotional and/or physical violence involved, and of course their immaturity, and being forced into child marriage. Consequently, often times married adolescent girls engage in sex as a duty to their husbands because they cannot refuse nor express their discontent. It is as if childhood marriage turns them into sexual slaves. In this study, some victims had to scream loudly as a coping strategy to draw the attention of their partners to their agony. When the noise is heard outside the bedroom by the in-laws, no emancipation comes, instead, sisters-in-law are tasked with counselling the married adolescent girls to be tolerant as the painful experience is short lived. This shows the role of culture in inculcating tolerance of sexual violence. The very people—sisters-in-law—who should have sympathized with their fellow female encourage her to persevere with an abusive and painful relationship. It appears there are no formal structures to which the married adolescent girls could turn to for support. Ineffective coping strategies against sexual violence in marriage have been reported elsewhere [23, 24, 41],

### The culture of silence about sexual violence

The married girls expressed a concern that they are culturally prepared to endure sexual violence in marriage. This implies that although they are essentially raped by their marital partners, they are expected not to speak out against the practice, instead, they should persevere. This culture of silence is inculcated through pre-marital teachings which instill feminine norms of sexual submissiveness. In many sub-Saharan African countries sexual submissiveness constitutes a key characteristic of a sexually ‘good’ woman [23]. This expectation is more so for married adolescent girls who occupy a much more subordinate position because of a huge age gap with their partners. Similar studies report that underreporting of sexual violence in marriage is rampant across Africa [44–47].

### Lack of protection by existing laws

A number of laws of Tanzania have been enacted in defense of women and children’s rights. Two of these are the Sexual Offences Special Provisions Act (SOSPA) number 4 of 1998 and the Law of Marriage Act of 1971 with 2012 Amendments [39, 48]. The SOSPA was enacted with the goal of protecting women and children from sexual violence. A critical examination of the Act, however, shows that vulnerability of adolescent brides to rape and forced sex was not well addressed, leaving them exposed to violent acts by their husbands.

In essence, this implies that adolescent girls are legally protected against rape so long as they are not married. The law also criminalizes sexual intercourse between a man and his spouse aged less than 15 years. Nevertheless, when the female spouse is 15 or more years of age, sexual intercourse with her “husband” is considered legal. This latter group is not protected by SOSPA. Consequently, they are left vulnerable to sexual attack and the abusers (husbands) are considered not guilty. Moreover, sections 10(2), 13(1) and 15 of Tanzania’s Marriage Act of 1971 allow marriage for 15-year-old girls, while the minimum age of marriage for boys is 18.

### Theoretical implications

Although the primary objective of this study was to articulate the experiences of sexual violence among married adolescent girls, the evidence has revealed that such experiences are shaped by broader social and cultural factors, the most prominent of these being the unequal power relations between an adolescent girl and her much older spouse. It is evident that young age compounds adolescent girls’ vulnerability to abuse. Thus, the descriptive data provided in this study could further the study on sexual violence in child marriages through the lens of Connell’s [43] and Wingood and DiClementi’s [49] works which identifies the profound inequalities created by three interlinked structures, namely: (a) the sexual division of labour; (b) the sexual division of power; and (c) the structure of cathexis (affective attachments and social norms). These structures exist both at family and societal levels, and are indoctrinated and perpetuated in society through socio-cultural norms.

### Study limitations

This article derives from a small-scale, qualitative study conducted in one district in Tanzania. The study generated rich information on the lived experiences of sexual violence among married adolescent girls, but care should be taken not to generalize beyond the local context. Importantly, there were limits to the extent to which this study explored married adolescent girls’ understanding and experience of early marriage and sex as a cultural and socially located practice. Furthermore, the study did not investigate the perspectives of the husbands. Future studies should consider addressing these gaps.

## Conclusion and recommendation

This study has policy and programmatic implications. The findings suggest that understanding married adolescent girls’ experiences of sexual violence is central to ensuring their concerns are properly integrated into gender-based violence policy and related control programs. In addition, there is a need for community-based programs to educate parents and guardians on negative impacts of child marriage. Lastly, the Tanzania Marriage Act of 1971 is overdue and should be revised to protect women and children. Given the study findings, efforts should be made to involve men, in-laws and community members to change the cultural and social norms that perpetuate. child marriages and consequent sexual violence. in Tanzania.

## Data Availability

Datasets used for this study are available from the corresponding author on reasonable request.

## Abbreviations

DHS: Demographic and Health Survey (DHS)
IPV: Intimate Partner Violence
NGO: Non-Governmental Organization (NGO)
SOSPA: Sexual Offences Special Provisions Act (SOSPA)
URT: United Republic of Tanzania

## References

[1] Chowdhury FD (2004). The Socio- cultural context of child marriage in a Bangladeshi village. Int J Soc Welf.

[2] Ayiga N, Rampage V (2013). Determinants of age at first marriage in sub Saharan Africa: a comparative study in Uganda and South Africa. J Soc Dev Afr.

[3] Birech J (2013). Child marriage: a cultural health phenomenon. Int J Human.

[4] The Government of the Republic of Zambia. Qualitative study of child marriage in six districts of Zambia. file:///C:/Users/User/Downloads/Qualitative%20study%20on%20Child%20Marriage%20in%20 Zambia%20(1).pdf (2015). Accessed 24 Nov 2018.

[5] Ministry of Health, Community Development, Gender, Elderly and Children, Tanzania (URT). National survey on the drivers and consequences of child marriage in Tanzania. http://www.cdftz.org/files/Forward%20230%20Page%20Report%202017%20WEB.pdf. Accessed 30 July 2018.

[6] Nour NM (2006). Health consequences of child marriage in Africa. Emerg Infect Dis.

[7] World Health Organization (WHO). Every woman, every child, every adolescent: achievements and prospects: The final report of the independent expert review group on information and accountability for women’s and children’s health. 2015. file:///C:/Users/Hp/AppData/Local/Packages/Microsoft.MicrosoftEdge_8wekyb3d8bbwe/TempState/Downloads/9789241509282_eng%20(1).pdf. Accessed 10 Apr 2020.

[8] Ellsberg M, Vyas A, Madrid B, Quintanilla M, Zelaya J, Stöckl H. Violence against adolescent girls: Falling through the cracks? Background paper. Ending violence in childhood Global Report 2017. Know Violence in Childhood. New Delhi, India. https://globalwomensinstitute.gwu.edu/sites/g/files/zaxdzs1356/f/downloads/Falling%20through%20the%20Cracks_Background%20Paper%20%281%29.pdf. Accessed 10 Aug 2019.

[9] Nove A, Matthews Z, Neal S, Camacho AV (2014). Maternal mortality in adolescents compared with women of other ages: evidence from 144 countries. Lancet Glob Health.

[10] Conde-Agudelo A, Belizán JM, Lammers C (2005). Maternal-perinatal morbidity and mortality associated with adolescent pregnancy in Latin America: cross-sectional study. Am J Obstet Gynecol.

[11] Belhorma S (2016). Two months of marriage were sufficient to turn my life upside down’: early marriage as a form of gender-based violence. Gender Dev.

[12] Deuba K, Mainali A, Alvesson HM, Karki DK (2016). Experience of intimate partner violence among young pregnant women in urban slums of Kathmandu Valley, Nepal: a qualitative study. BMC Womens Health..

[13] Abdool KQ, Baxter C (2016). The dual burden of gender-based violence and HIV in adolescent girls and young women in South Africa. S Afr Med J.

[14] Ghanotakis EMayhew S, Watts C (2009). Tackling HIV and gender-based violence in South Africa: how has PEPFAR responded and what are the implications for implementing organizations?. Health Policy Plan.

[15] Andersson N, Cockcroft A, Shea B (2008). Gender-based violence and HIV: relevance for HIV prevention in hyperendemic countries of southern Africa. AIDS Suppl.

[16] Durevall D, Lindskog A (2015). Intimate partner violence and HIV in ten sub-Saharan African countries: what do the Demographic and Health Surveys tell us?. Lancet Glob Health.

[17] Vergaede S. Intimate partner violence in Malawi: Master’s Dissertation, Universiteit Gent, 2016.

[18] World Health Organization (WHO) (2005). Multi-country study on women’s health and domestic violence against women: initial results on prevalence, health outcomes, and women’s responses.

[19] McCloskey LA, Williams C, Larsen U (2005). Gender inequality and intimate partner violence among women in Moshi. Tanzania In Fam Plan Perspect.

[20] Stöckl H, March L, Pallitto C, Garcia-Moreno C (2014). Intimate partner violence among adolescents and young women: prevalence and associated factors in nine countries: a cross sectional study. BMC Public Health..

[21] Kapiga S, Harvey S, Muhammad AK, Stocki H, Mshana G, Hashim R, Hansen C, Lees S, Watts C (2017). Prevalence of intimate partner violence and abuse and associated factors among women enrolled into a cluster randomised trial in northwestern Tanzania. BMC Public Health..

[22] Kazaura MR, Ezekiel MJ, Chitama D (2016). Magnitude and factors associated with intimate partner violence in mainland Tanzania. BMC Public Health..

[23] Nyamhanga T, Frumence G (2014). Gender context of sexual violence and HIV sexual risk behaviors among married women in Iringa Region. Tanzania. Glob Health Action..

[24] Puri M, Tamang I, Shah I (2011). Suffering in silence: consequences of sexual violence within marriage among young women in Nepal. BMC Public Health.

[25] Erulkar A (2013). Early marriage, marital relations and intimate partner violence in Ethiopia. Int Perspect Sex Reprod Health.

[26] Falb KL, Annan J, Kpebo D, Cole H, Willie T, Xuan Z, Raj A, Gupta J (2015). Differential impacts of an intimate partner violence prevention program based on child marriage status in rural Côte d’Ivoire. J Adolesc Health.

[27] Peterman A, Bleck J, Palemo T (2015). Age and intimate partner violence: An analysis of global trends among women experiencing victimization in 30 developing countries. J Adolesc Health.

[28] UNFPA Tanzania. Fact sheet: Child marriage. https://tanzania.unfpa.org/sites/default/files/pub-pdf/Factsheet_CM_UNFPA_14oct.pdf. 2018. Accessed 05 Feb 2019.

[29] Santhya KG (2011). Early marriage and sexual and reproductive health vulnerabilities of young women: a synthesis of recent evidence from developing countries. Curr Opin Obstet Gynecol.

[30] Raj A (2010). When the mother is a child: the impact of child marriage on the health and human rights of girls. Arch Dis Child.

[31] Petroni S, Steinhaus M, Fenn NS, Stoebenau K, Gregowski A (2017). New findings on child marriage in sub-Saharan Africa. Ann Glob Health.

[32] MwanukuziChild marriage: Lived experiences of sexual and reproductive health in Kahama, Shinyanga, Tanzania. Unpublished MPH Diss., Muhimbili University of Health and Allied Sciences. 2015.

[33] Ministry of Health, Community Development, Gender, Elderly and Children (MoHCDGEC). Child marriage in Tanzania at glance. Dar es Salaam: MoHCDGEC, 2017.

[34] Guest G, Bunce A, Johnson L (2006). How many interviews are enough? An experiment with data saturation and variability. Field Methods.

[35] Dahlgren L, Emmelin M, Winkvist A (2004). Qualitative methodology in international public health.

[36] Sandelowski M (1985). Sample size in qualitative research. Res Nurs Health.

[37] Attride-Stirling J (2001). Thematic networks: an analytical tool for qualitative research. Qual Res J.

[38] Thomas J, Harden A (2008). Methods for the thematic synthesis of qualitative research in systematic reviews. BMC Med Res Methodol..

[39] Lincoln YS, Guba EG (1985). Naturalistic inquiry.

[40] McCloskey LA, Williams C, Larse U (2005). Gender inequality and intimate partner violence among women in Moshi. Tanzania Int Fam Plan Perspect.

[41] Cáceres C, Jejeebhoy S, Shah I, Thapa S (2005). Assessing young people’s non-consensual sexual experiences: lessons from Peru. Sex without consent: Young people in developing countries.

[42] Connell RW (1987). Gender and power: society, the person, and sexual politics.

[43] Olukemi AA, Folakemi OC. Culture of silence and wave of sexual violence in Nigeria. AASCIT Journal of Education. 2015;1(3):31–37. http://www.aascit.org/journal/education.

[44] Akinlusi FM, Rabiu AK, Olawepo TA, Adewumi AA, Ottun AT, Akinola OI (2014). Sexual assault in Lagos, Nigeria: a five year retrospective review. BMC Womens Health..

[45] Indupalli AS, Giri PA (2014). Sexual violence among married women: an unspoken sting. J Res Med Sci.

[46] United Republic of Tanzania (URT). Sexual Offences Special Provisions Act No. 4 of 1998. http://crm.misa.org/upload/web/SOSPA%204-1998.pdf. Accessed 07 Sept 2018.

[47] United Republic of Tanzania (URT). The Law of Marriage Act No. 5 of 1971. http://www.rita.go.tz/eng/laws/History%20Laws/Marriage%20Ordinance,%20(cap%2029).pdf. Accessed 07 Sept 2018.

[48] Wingood GM, DiClementi RJ (2000). Application of the theory of gender and power to examine HIV-related exposures, risk factors, and effective interventions for women. Health Educ Behav.

[49] Niec A (2002). Forensic issues in the assessment of sexually assaulted adolescents. Paediatr Child Health.

